# 6-Butyl-5-(4-methoxy­phen­oxy)-3-phenyl-3*H*-1,2,3-triazolo[4,5-*d*]pyrimidin-7(6*H*)-one

**DOI:** 10.1107/S1600536809046017

**Published:** 2009-11-07

**Authors:** Xiao-Hua Zeng, Shou-Heng Deng, Ping Chen, Hong-Mei Wang, Zuan Ma

**Affiliations:** a Institute of Medicinal Chemistry, Yunyang Medical College, Shiyan 442000, People’s Republic of China; b Center of Oncology, People’s Hospital Affiliated with Yunyang Medical College, Shiyan 442000, People’s Republic of China

## Abstract

The asymmetric unit of the title compound, C_21_H_21_N_5_O_3_, consists of two geometrically similar mol­ecules. The fused rings of the triazolo[4,5-*d*]pyrimidine system are nearly coplanar, making dihedral angels of 1.48 (18) and 1.34 (16)°, and the phenyl rings are twisted by 12.3 (1) and 8.7 (1)° with respect to the triazolopyrimidine plane. The ethyl groups of the *n*-butyl side chains are disordered over two sites in each of the independent mol­ecules, the ratios of occupancies being 0.60:0.40 and 0.61:0.39.

## Related literature

For the biological activity of 8-aza­guanine derivatives, see: Roblin *et al.* (1945 ▶); Ding *et al.* (2004 ▶); Mitchell *et al.* (1950 ▶); Levine *et al.* (1963 ▶); Montgomery *et al.* (1962 ▶)); Yamamoto *et al.* (1967 ▶); Bariana (1971 ▶); Holland *et al.* (1975 ▶). For related structures, see: Wang *et al.* (2006 ▶); Zeng *et al.* (2006 ▶, 2009 ▶); Zhao, Hu *et al.* (2005 ▶); Zhao, Wang & Ding (2005 ▶); Zhao, Xie *et al.* (2005 ▶).
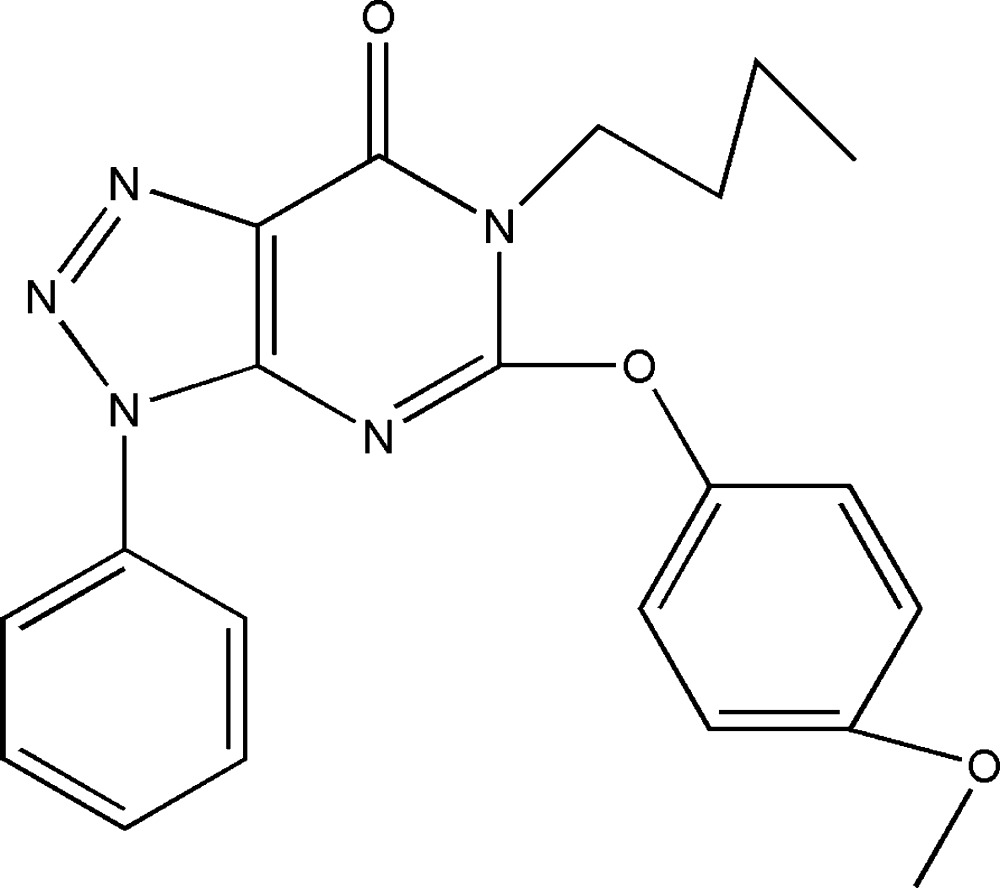


## Experimental

### 

#### Crystal data

C_21_H_21_N_5_O_3_
*M* *_r_* = 391.43
Triclinic, 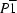

*a* = 11.5167 (13) Å
*b* = 12.4026 (13) Å
*c* = 14.9353 (16) Å
α = 78.795 (2)°
β = 76.207 (2)°
γ = 76.360 (2)°
*V* = 1991.7 (4) Å^3^
*Z* = 4
Mo *K*α radiation
μ = 0.09 mm^−1^
*T* = 298 K
0.15 × 0.12 × 0.10 mm

#### Data collection

Bruker SMART CCD area-detector diffractometer
Absorption correction: multi-scan (*SADABS*; Sheldrick, 1996 ▶) *T*_min_ = 0.987, *T*_max_ = 0.991
11898 measured reflections
6940 independent reflections
5252 reflections with *I* > 2σ(*I*)
*R*_int_ = 0.061

#### Refinement

*R*[*F* ^2^ > 2σ(*F* ^2^)] = 0.079
*wR*(*F* ^2^) = 0.212
*S* = 1.07
6940 reflections
567 parameters
12 restraints
H-atom parameters constrained
Δρ_max_ = 0.44 e Å^−3^
Δρ_min_ = −0.22 e Å^−3^

### 

Data collection: *SMART* (Bruker, 2001 ▶); cell refinement: *SAINT* (Bruker, 2001 ▶); data reduction: *SAINT*; program(s) used to solve structure: *SHELXS97* (Sheldrick, 2008 ▶); program(s) used to refine structure: *SHELXL97* (Sheldrick, 2008 ▶); molecular graphics: *PLATON* (Spek, 2009 ▶); software used to prepare material for publication: *SHELXTL97* (Sheldrick, 2008 ▶).

## Supplementary Material

Crystal structure: contains datablocks global, I. DOI: 10.1107/S1600536809046017/ya2108sup1.cif

Structure factors: contains datablocks I. DOI: 10.1107/S1600536809046017/ya2108Isup2.hkl

Additional supplementary materials:  crystallographic information; 3D view; checkCIF report

## Figures and Tables

**Fig. 1.**
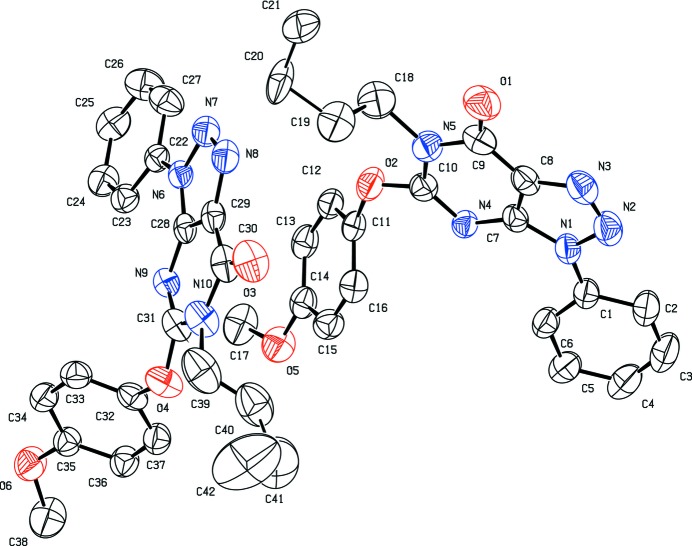
The asymmetric unit of the title compound with atomic numbering scheme and displacement ellipsoids drawn at the 30% probability level; only major components of disordered butyl groups are shown. H-Atoms are omitted.

## supplementary crystallographic information

### Comment

The derivatives of heterocycles containing 8-azaguanine system, which are well known bioisosteres of guanine, are of great importance because of their remarkable biological properties, such as antimicrobial or antifungal activities (Roblin *et al.*, 1945; Ding *et al.*, 2004), encephaloma cell inhibitor (Mitchell *et al.*, 1950; Levine *et al.*, 1963), antileukemia (Montgomery *et al.*, 1962), hypersusceptibility inhibitor and acesodyne activities (Yamamoto *et al.*, 1967; Bariana, 1971; Holland *et al.*, 1975).

In recent years, Zhao's group succeeded in synthesizing the derivatives of 8-azaguanine *via* aza-Wittig reaction of beta-ethoxycarbonyl iminophosphorane with aromatic isocyanates (Zhao, Xie *et al.*, 2005). As a continuation of the quest for new biologically active derivatives of 8-azaguanine, the title compound was obtained from beta-ethoxycarbonyl iminophosphorane and aliphatic isocyanate and structurally characterized.

The asymmetric unit of the crystal of the title compound, C_21_H_21_N_5_O_3_, consists of two geometrically similar molecules (Fig. 1), the bond lengths and angles in triazolopyrimidinone moiety are in good agreement with those observed for a closely related structures (Zhao, Hu *et al.*, 2005; Zhao, Wang & Ding, 2005). The fused rings of the triazolo[4,5-*d*]pyrimidine ring system are as usual coplanar (e.g. see structures by Wang *et al.*, 2006 and Zeng *et al.*, 2009), and the phenyl rings are twisted with respect to the triazolopyrimidine plane by 12.3 (1)° and 8.7 (1)° in each of the two independent molecules.

### Experimental

To the solution of carbodiimide, prepared according to Zeng *et al.* (2006) in a mixed solvent (CH_2_Cl_2_/CH_3_CN, 1:4 *v*/*v*, 15 ml), was added 4-chlorophenol (3 mmol) and K_2_CO_3_ (6 mmol). After the reaction mixture was stirred for 12 h, the solvent was removed under reduced pressure and the residue was recrystallized from EtOH to give the title compound in 93% yield (m.p. 426 K). Elemental analysis: calculated for C_21_H_21_N_5_O_3_: C, 64.44; H, 5.41; N, 17.89%. Found: C, 63.62; H, 5.68; N, 17.51%. Crystals suitable for X-ray diffraction study were obtained by recrystallization from hexane and dichloromethane (1:3 *v*/*v*) at room temperature.

### Refinement

All H atoms were placed in the geometrically calculated positions and treated as riding atoms, with C—H = 0.93–0.97 Å, and *U*_iso_(H) = 1.2*U*_eq_(C) [1.5*U*_eq_(C) for methyl H atoms]. The terminal ethyl groups of the n-butyl substituent are disordered in both molecules; the occupancy refinement yielded ratio 0.60 : 0.40 and 0.61 : 0.39 for each of the two molecules. The bond lengths and the 1,3-distances in these groups were restrained during the refinement to be within the ranges of 1,54 ± 0.01 Å and 2.45 ± 0.01 Å respectively.

### Crystal data

**Table:**

C_21_H_21_N_5_O_3_	*Z* = 4
*M**_r_* = 391.43	*F*(000) = 824
Triclinic, *P*1	*D*_x_ = 1.305 Mg m^−^^3^
Hall symbol: -P 1	Mo *K*α radiation, λ = 0.71073 Å
*a* = 11.5167 (13) Å	Cell parameters from 4234 reflections
*b* = 12.4026 (13) Å	θ = 2.3–25.4°
*c* = 14.9353 (16) Å	µ = 0.09 mm^−^^1^
α = 78.795 (2)°	*T* = 298 K
β = 76.207 (2)°	Block, colorless
γ = 76.360 (2)°	0.15 × 0.12 × 0.10 mm
*V* = 1991.7 (4) Å^3^	

### Data collection

**Table:**

Bruker SMART CCD area-detector diffractometer	6940 independent reflections
Radiation source: fine-focus sealed tube	5252 reflections with *I* > 2σ(*I*)
graphite	*R*_int_ = 0.061
φ and ω scans	θ_max_ = 25.0°, θ_min_ = 2.0°
Absorption correction: multi-scan (*SADABS*; Sheldrick, 1996)	*h* = −13→13
*T*_min_ = 0.987, *T*_max_ = 0.991	*k* = −13→14
11898 measured reflections	*l* = −17→17

### Refinement

**Table:**

Refinement on *F*^2^	Primary atom site location: structure-invariant direct methods
Least-squares matrix: full	Secondary atom site location: difference Fourier map
*R*[*F*^2^ > 2σ(*F*^2^)] = 0.079	Hydrogen site location: inferred from neighbouring sites
*wR*(*F*^2^) = 0.212	H-atom parameters constrained
*S* = 1.07	*w* = 1/[σ^2^(*F*_o_^2^) + (0.0878*P*)^2^ + 0.7377*P*] where *P* = (*F*_o_^2^ + 2*F*_c_^2^)/3
6940 reflections	(Δ/σ)_max_ < 0.001
567 parameters	Δρ_max_ = 0.44 e Å^−^^3^
12 restraints	Δρ_min_ = −0.22 e Å^−^^3^

### Special details

**Table:**

Geometry. All e.s.d.'s (except the e.s.d. in the dihedral angle between two l.s. planes) are estimated using the full covariance matrix. The cell e.s.d.'s are taken into account individually in the estimation of e.s.d.'s in distances, angles and torsion angles; correlations between e.s.d.'s in cell parameters are only used when they are defined by crystal symmetry. An approximate (isotropic) treatment of cell e.s.d.'s is used for estimating e.s.d.'s involving l.s. planes.
Refinement. Refinement of *F*^2^ against ALL reflections. The weighted *R*-factor *wR* and goodness of fit *S* are based on *F*^2^, conventional *R*-factors *R* are based on *F*, with *F* set to zero for negative *F*^2^. The threshold expression of *F*^2^ > σ(*F*^2^) is used only for calculating *R*-factors(gt) *etc*. and is not relevant to the choice of reflections for refinement. *R*-factors based on *F*^2^ are statistically about twice as large as those based on *F*, and *R*- factors based on ALL data will be even larger.

### Fractional atomic coordinates and isotropic or equivalent isotropic displacement parameters (Å^2^)

**Table:**

	*x*	*y*	*z*	*U*_iso_*/*U*_eq_	Occ. (<1)
C1	0.3813 (3)	1.1282 (3)	−0.0630 (2)	0.0704 (8)	
C2	0.4159 (3)	1.2053 (3)	−0.1390 (3)	0.0929 (11)	
H2	0.4473	1.2648	−0.1315	0.112*	
C3	0.4038 (4)	1.1937 (4)	−0.2255 (3)	0.1134 (15)	
H3	0.4286	1.2451	−0.2767	0.136*	
C4	0.3560 (4)	1.1082 (4)	−0.2378 (3)	0.1053 (13)	
H4	0.3469	1.1021	−0.2968	0.126*	
C5	0.3215 (3)	1.0312 (3)	−0.1625 (3)	0.0918 (10)	
H5	0.2897	0.9721	−0.1703	0.110*	
C6	0.3341 (3)	1.0418 (3)	−0.0745 (2)	0.0789 (9)	
H6	0.3103	0.9900	−0.0233	0.095*	
C7	0.3573 (3)	1.0900 (2)	0.1139 (2)	0.0642 (7)	
C8	0.4017 (3)	1.1368 (3)	0.1700 (3)	0.0748 (8)	
C9	0.3731 (3)	1.1058 (3)	0.2691 (3)	0.0856 (10)	
C10	0.2607 (3)	0.9846 (3)	0.2292 (2)	0.0708 (8)	
C11	0.1502 (3)	0.8491 (3)	0.2166 (2)	0.0695 (8)	
C12	0.0289 (3)	0.8462 (3)	0.2362 (2)	0.0759 (9)	
H12	−0.0257	0.8930	0.2758	0.091*	
C13	−0.0129 (3)	0.7744 (3)	0.1976 (2)	0.0775 (9)	
H13	−0.0955	0.7721	0.2110	0.093*	
C14	0.0688 (3)	0.7057 (3)	0.1388 (2)	0.0732 (8)	
C15	0.1902 (3)	0.7121 (3)	0.1176 (2)	0.0810 (9)	
H15	0.2447	0.6676	0.0761	0.097*	
C16	0.2326 (3)	0.7834 (3)	0.1569 (2)	0.0767 (9)	
H16	0.3149	0.7867	0.1432	0.092*	
C17	−0.0777 (4)	0.6012 (4)	0.1313 (3)	0.1118 (14)	
H17A	−0.0869	0.5741	0.1969	0.168*	
H17B	−0.0848	0.5438	0.0993	0.168*	
H17C	−0.1401	0.6663	0.1212	0.168*	
C18	0.2467 (5)	0.9939 (4)	0.3997 (3)	0.1173 (14)	
H18A	0.2351	1.0582	0.4312	0.141*	
H18B	0.1682	0.9732	0.4084	0.141*	
C19	0.3312 (5)	0.9008 (5)	0.4395 (3)	0.1241 (15)	
H19A	0.4072	0.9247	0.4342	0.149*	0.60
H19B	0.3485	0.8401	0.4030	0.149*	0.60
H19C	0.4133	0.9143	0.4292	0.149*	0.40
H19D	0.3321	0.8293	0.4218	0.149*	0.40
C20	0.2846 (11)	0.8550 (9)	0.5437 (5)	0.121 (4)	0.60
H20A	0.2018	0.8435	0.5531	0.145*	0.60
H20B	0.3363	0.7840	0.5619	0.145*	0.60
C21	0.2888 (10)	0.9402 (8)	0.5999 (5)	0.132 (3)	0.60
H21A	0.3635	0.9671	0.5768	0.198*	0.60
H21B	0.2846	0.9068	0.6639	0.198*	0.60
H21C	0.2209	1.0016	0.5952	0.198*	0.60
C22	−0.1449 (3)	0.6338 (2)	0.43768 (18)	0.0621 (7)	
C23	−0.1744 (3)	0.5483 (3)	0.4068 (2)	0.0720 (8)	
H23	−0.1134	0.4941	0.3790	0.086*	
C24	−0.2963 (3)	0.5440 (3)	0.4179 (2)	0.0820 (9)	
H24	−0.3169	0.4866	0.3967	0.098*	
C25	−0.3869 (3)	0.6231 (4)	0.4594 (2)	0.0891 (10)	
H25	−0.4685	0.6199	0.4663	0.107*	
C26	−0.3555 (4)	0.7063 (4)	0.4904 (3)	0.1013 (12)	
H26	−0.4164	0.7599	0.5191	0.122*	
C27	−0.2362 (4)	0.7126 (3)	0.4800 (3)	0.0895 (10)	
H27	−0.2165	0.7701	0.5016	0.107*	
C28	0.0845 (3)	0.5873 (2)	0.37838 (19)	0.0593 (7)	
C29	0.1745 (3)	0.6332 (3)	0.3916 (2)	0.0678 (8)	
C30	0.2988 (3)	0.5944 (3)	0.3509 (3)	0.0815 (10)	
C31	0.2108 (3)	0.4696 (3)	0.2899 (2)	0.0733 (8)	
C32	0.1543 (3)	0.3377 (3)	0.2199 (2)	0.0700 (8)	
C33	0.0893 (3)	0.2762 (3)	0.2924 (2)	0.0785 (9)	
H33	0.0932	0.2781	0.3536	0.094*	
C34	0.0188 (3)	0.2120 (3)	0.2740 (2)	0.0786 (9)	
H34	−0.0263	0.1710	0.3232	0.094*	
C35	0.0135 (3)	0.2071 (3)	0.1841 (2)	0.0701 (8)	
C36	0.0764 (3)	0.2719 (3)	0.1126 (2)	0.0783 (9)	
H36	0.0713	0.2714	0.0515	0.094*	
C37	0.1466 (3)	0.3373 (3)	0.1306 (2)	0.0781 (9)	
H37	0.1887	0.3812	0.0818	0.094*	
C38	−0.0400 (5)	0.1100 (4)	0.0814 (3)	0.1248 (17)	
H38A	−0.0841	0.1719	0.0454	0.187*	
H38B	−0.0717	0.0439	0.0850	0.187*	
H38C	0.0448	0.0972	0.0521	0.187*	
C39	0.4370 (5)	0.4412 (4)	0.2681 (5)	0.1387 (19)	
H39A	0.4898	0.4463	0.3083	0.166*	
H39B	0.4363	0.3626	0.2704	0.166*	
C40	0.4816 (4)	0.4881 (4)	0.1756 (5)	0.1389 (19)	
H40A	0.4737	0.5671	0.1781	0.167*	0.61
H40B	0.4220	0.4841	0.1407	0.167*	0.61
H40C	0.4748	0.5685	0.1698	0.167*	0.39
H40D	0.4417	0.4716	0.1310	0.167*	0.39
C41	0.6105 (11)	0.4531 (16)	0.1097 (10)	0.266 (13)	0.61
H41A	0.6056	0.4016	0.0701	0.319*	0.61
H41B	0.6361	0.5188	0.0703	0.319*	0.61
C42	0.6979 (10)	0.3988 (19)	0.1703 (12)	0.284 (11)	0.61
H42A	0.7216	0.4550	0.1942	0.427*	0.61
H42B	0.7687	0.3562	0.1350	0.427*	0.61
H42C	0.6605	0.3497	0.2212	0.427*	0.61
C20'	0.2534 (19)	0.9186 (14)	0.5406 (7)	0.208 (14)	0.40
H20C	0.2578	0.9900	0.5557	0.250*	0.40
H20D	0.1686	0.9167	0.5446	0.250*	0.40
C21'	0.3110 (14)	0.8215 (16)	0.6063 (8)	0.170 (7)	0.40
H21D	0.3036	0.7518	0.5914	0.255*	0.40
H21E	0.2700	0.8287	0.6694	0.255*	0.40
H21F	0.3957	0.8229	0.5992	0.255*	0.40
C41'	0.6174 (7)	0.4221 (14)	0.1683 (10)	0.131 (6)	0.39
H41C	0.6526	0.4362	0.2167	0.157*	0.39
H41D	0.6214	0.3421	0.1743	0.157*	0.39
C42'	0.6838 (12)	0.4652 (15)	0.0738 (11)	0.174 (8)	0.39
H42D	0.6476	0.4508	0.0268	0.261*	0.39
H42E	0.7680	0.4279	0.0651	0.261*	0.39
H42F	0.6785	0.5445	0.0689	0.261*	0.39
N1	0.3984 (2)	1.1397 (2)	0.0256 (2)	0.0718 (7)	
N2	0.4667 (3)	1.2151 (2)	0.0314 (3)	0.0924 (9)	
N3	0.4686 (3)	1.2126 (2)	0.1183 (3)	0.0922 (9)	
N4	0.2861 (2)	1.0120 (2)	0.13914 (17)	0.0651 (6)	
N5	0.2979 (3)	1.0252 (2)	0.29391 (19)	0.0853 (8)	
N6	−0.0203 (2)	0.64224 (19)	0.42687 (15)	0.0627 (6)	
N7	0.0072 (3)	0.7202 (2)	0.46881 (18)	0.0786 (8)	
N8	0.1237 (3)	0.7147 (2)	0.44767 (18)	0.0818 (8)	
N9	0.0976 (2)	0.50531 (19)	0.32598 (16)	0.0607 (6)	
N10	0.3093 (2)	0.5042 (2)	0.3012 (2)	0.0888 (9)	
O1	0.4041 (3)	1.1411 (2)	0.3284 (2)	0.1146 (9)	
O2	0.1887 (2)	0.9109 (2)	0.26967 (15)	0.0907 (7)	
O3	0.3883 (2)	0.6278 (2)	0.3538 (2)	0.1099 (9)	
O4	0.2423 (2)	0.3919 (2)	0.23281 (19)	0.0926 (7)	
O5	0.0382 (2)	0.6299 (2)	0.09702 (18)	0.0975 (8)	
O6	−0.0535 (2)	0.1350 (2)	0.17288 (18)	0.0985 (8)	

### Atomic displacement parameters (Å^2^)

**Table:**

	*U*^11^	*U*^22^	*U*^33^	*U*^12^	*U*^13^	*U*^23^
C1	0.0525 (16)	0.0621 (19)	0.080 (2)	−0.0022 (14)	−0.0041 (14)	0.0059 (16)
C2	0.083 (2)	0.082 (2)	0.095 (3)	−0.0164 (19)	−0.0054 (19)	0.016 (2)
C3	0.103 (3)	0.118 (4)	0.089 (3)	−0.017 (3)	−0.004 (2)	0.031 (3)
C4	0.088 (3)	0.134 (4)	0.071 (2)	−0.006 (3)	−0.0051 (19)	0.006 (2)
C5	0.086 (2)	0.104 (3)	0.075 (2)	−0.012 (2)	−0.0074 (18)	−0.008 (2)
C6	0.069 (2)	0.080 (2)	0.072 (2)	−0.0116 (17)	−0.0004 (15)	0.0038 (17)
C7	0.0537 (16)	0.0508 (16)	0.079 (2)	−0.0017 (13)	−0.0093 (14)	−0.0045 (15)
C8	0.0682 (19)	0.0572 (18)	0.096 (2)	−0.0068 (15)	−0.0154 (17)	−0.0129 (17)
C9	0.092 (2)	0.073 (2)	0.098 (3)	−0.0042 (19)	−0.031 (2)	−0.026 (2)
C10	0.0747 (19)	0.0667 (19)	0.070 (2)	−0.0118 (16)	−0.0160 (15)	−0.0097 (16)
C11	0.085 (2)	0.0651 (18)	0.0525 (16)	−0.0167 (17)	−0.0152 (15)	0.0094 (14)
C12	0.072 (2)	0.071 (2)	0.0683 (19)	−0.0076 (16)	−0.0023 (15)	0.0062 (16)
C13	0.0637 (19)	0.084 (2)	0.075 (2)	−0.0144 (17)	−0.0173 (16)	0.0143 (18)
C14	0.072 (2)	0.074 (2)	0.0682 (19)	−0.0107 (17)	−0.0227 (16)	0.0077 (16)
C15	0.074 (2)	0.084 (2)	0.078 (2)	−0.0077 (18)	−0.0110 (16)	−0.0093 (18)
C16	0.0640 (19)	0.080 (2)	0.082 (2)	−0.0157 (17)	−0.0182 (16)	0.0023 (18)
C17	0.113 (3)	0.139 (4)	0.100 (3)	−0.057 (3)	−0.036 (2)	0.002 (3)
C18	0.135 (4)	0.123 (4)	0.108 (3)	−0.033 (3)	−0.042 (3)	−0.021 (3)
C19	0.124 (4)	0.139 (4)	0.119 (4)	−0.041 (3)	−0.033 (3)	−0.011 (3)
C20	0.171 (8)	0.147 (10)	0.065 (5)	−0.097 (7)	−0.039 (5)	0.034 (5)
C21	0.197 (9)	0.137 (8)	0.071 (5)	−0.052 (7)	−0.016 (5)	−0.029 (5)
C22	0.082 (2)	0.0595 (17)	0.0467 (14)	−0.0145 (15)	−0.0234 (13)	0.0009 (13)
C23	0.081 (2)	0.075 (2)	0.0632 (18)	−0.0214 (17)	−0.0150 (15)	−0.0092 (15)
C24	0.089 (2)	0.094 (2)	0.073 (2)	−0.032 (2)	−0.0205 (18)	−0.0121 (18)
C25	0.075 (2)	0.116 (3)	0.075 (2)	−0.011 (2)	−0.0279 (18)	−0.006 (2)
C26	0.088 (3)	0.105 (3)	0.112 (3)	0.010 (2)	−0.038 (2)	−0.033 (2)
C27	0.104 (3)	0.079 (2)	0.096 (3)	−0.007 (2)	−0.040 (2)	−0.027 (2)
C28	0.0784 (19)	0.0533 (16)	0.0523 (15)	−0.0204 (15)	−0.0306 (14)	0.0081 (13)
C29	0.090 (2)	0.0656 (18)	0.0599 (17)	−0.0326 (17)	−0.0351 (16)	0.0073 (15)
C30	0.086 (2)	0.078 (2)	0.097 (2)	−0.0317 (19)	−0.049 (2)	0.0062 (18)
C31	0.068 (2)	0.0630 (19)	0.097 (2)	−0.0120 (15)	−0.0364 (17)	−0.0077 (17)
C32	0.0551 (17)	0.0622 (18)	0.093 (2)	−0.0032 (14)	−0.0152 (15)	−0.0213 (17)
C33	0.087 (2)	0.078 (2)	0.071 (2)	−0.0079 (18)	−0.0181 (17)	−0.0196 (17)
C34	0.086 (2)	0.081 (2)	0.069 (2)	−0.0279 (18)	−0.0039 (16)	−0.0120 (17)
C35	0.0668 (18)	0.0706 (19)	0.073 (2)	−0.0176 (15)	−0.0114 (15)	−0.0085 (16)
C36	0.089 (2)	0.083 (2)	0.0656 (19)	−0.0233 (19)	−0.0201 (16)	−0.0038 (17)
C37	0.076 (2)	0.074 (2)	0.080 (2)	−0.0204 (17)	−0.0094 (16)	−0.0014 (17)
C38	0.166 (5)	0.150 (4)	0.093 (3)	−0.082 (4)	−0.041 (3)	−0.017 (3)
C39	0.111 (4)	0.105 (4)	0.219 (7)	−0.035 (3)	−0.059 (4)	−0.020 (4)
C40	0.109 (4)	0.106 (4)	0.202 (6)	−0.027 (3)	−0.032 (4)	−0.015 (4)
C41	0.162 (15)	0.182 (13)	0.32 (3)	0.038 (11)	0.090 (15)	0.014 (17)
C42	0.108 (9)	0.44 (3)	0.254 (19)	0.042 (14)	−0.034 (11)	−0.048 (19)
C20'	0.41 (4)	0.164 (19)	0.071 (10)	−0.15 (2)	−0.052 (14)	0.041 (11)
C21'	0.121 (11)	0.22 (2)	0.132 (13)	−0.017 (12)	0.003 (9)	0.008 (13)
C41'	0.044 (6)	0.194 (18)	0.154 (13)	−0.035 (8)	−0.026 (7)	−0.002 (11)
C42'	0.100 (11)	0.192 (16)	0.205 (18)	−0.034 (11)	0.076 (12)	−0.095 (15)
N1	0.0600 (15)	0.0578 (15)	0.0877 (19)	−0.0113 (12)	−0.0045 (13)	−0.0002 (13)
N2	0.082 (2)	0.0746 (19)	0.115 (3)	−0.0258 (16)	−0.0075 (17)	−0.0036 (18)
N3	0.085 (2)	0.0727 (19)	0.121 (3)	−0.0197 (16)	−0.0201 (18)	−0.0163 (18)
N4	0.0657 (15)	0.0622 (15)	0.0607 (15)	−0.0086 (12)	−0.0096 (11)	−0.0021 (11)
N5	0.102 (2)	0.0855 (19)	0.0711 (18)	−0.0220 (17)	−0.0203 (15)	−0.0098 (15)
N6	0.0871 (18)	0.0588 (14)	0.0496 (13)	−0.0233 (13)	−0.0235 (12)	−0.0023 (11)
N7	0.111 (2)	0.0760 (17)	0.0627 (15)	−0.0389 (16)	−0.0237 (15)	−0.0095 (13)
N8	0.117 (2)	0.0825 (19)	0.0650 (16)	−0.0486 (18)	−0.0337 (15)	−0.0017 (14)
N9	0.0634 (15)	0.0572 (14)	0.0666 (14)	−0.0112 (11)	−0.0280 (11)	−0.0040 (11)
N10	0.0599 (16)	0.0815 (19)	0.134 (3)	−0.0122 (14)	−0.0391 (16)	−0.0159 (18)
O1	0.144 (3)	0.103 (2)	0.114 (2)	−0.0306 (18)	−0.0407 (18)	−0.0324 (17)
O2	0.1180 (19)	0.0982 (17)	0.0616 (13)	−0.0480 (16)	−0.0136 (12)	0.0013 (12)
O3	0.0943 (18)	0.114 (2)	0.147 (2)	−0.0462 (16)	−0.0537 (17)	−0.0124 (18)
O4	0.0615 (13)	0.0880 (16)	0.138 (2)	−0.0121 (12)	−0.0179 (13)	−0.0449 (16)
O5	0.0979 (18)	0.1090 (19)	0.0956 (17)	−0.0366 (15)	−0.0251 (14)	−0.0123 (15)
O6	0.1103 (19)	0.1090 (19)	0.0899 (17)	−0.0546 (16)	−0.0113 (14)	−0.0179 (14)

### Geometric parameters (Å, °)

**Table:**

C1—C6	1.366 (4)	C25—H25	0.9300
C1—C2	1.379 (5)	C26—C27	1.364 (5)
C1—N1	1.422 (4)	C26—H26	0.9300
C2—C3	1.370 (6)	C27—H27	0.9300
C2—H2	0.9300	C28—N6	1.354 (4)
C3—C4	1.365 (6)	C28—N9	1.360 (4)
C3—H3	0.9300	C28—C29	1.363 (4)
C4—C5	1.373 (5)	C29—N8	1.373 (4)
C4—H4	0.9300	C29—C30	1.422 (5)
C5—C6	1.390 (5)	C30—O3	1.211 (4)
C5—H5	0.9300	C30—N10	1.426 (4)
C6—H6	0.9300	C31—N9	1.293 (4)
C7—C8	1.354 (4)	C31—O4	1.339 (4)
C7—N4	1.355 (4)	C31—N10	1.359 (4)
C7—N1	1.366 (4)	C32—C37	1.359 (5)
C8—N3	1.360 (4)	C32—C33	1.371 (5)
C8—C9	1.432 (5)	C32—O4	1.407 (4)
C9—O1	1.217 (4)	C33—C34	1.365 (4)
C9—N5	1.413 (5)	C33—H33	0.9300
C10—N4	1.301 (4)	C34—C35	1.373 (4)
C10—O2	1.339 (4)	C34—H34	0.9300
C10—N5	1.363 (4)	C35—O6	1.367 (4)
C11—C12	1.365 (4)	C35—C36	1.371 (4)
C11—C16	1.368 (5)	C36—C37	1.372 (4)
C11—O2	1.406 (4)	C36—H36	0.9300
C12—C13	1.376 (5)	C37—H37	0.9300
C12—H12	0.9300	C38—O6	1.424 (5)
C13—C14	1.379 (5)	C38—H38A	0.9600
C13—H13	0.9300	C38—H38B	0.9600
C14—O5	1.374 (4)	C38—H38C	0.9600
C14—C15	1.376 (4)	C39—C40	1.414 (8)
C15—C16	1.382 (5)	C39—N10	1.510 (6)
C15—H15	0.9300	C39—H39A	0.9700
C16—H16	0.9300	C39—H39B	0.9700
C17—O5	1.413 (4)	C40—C41'	1.574 (9)
C17—H17A	0.9600	C40—C41	1.590 (9)
C17—H17B	0.9600	C40—H40A	0.9700
C17—H17C	0.9600	C40—H40B	0.9700
C18—C19	1.454 (6)	C40—H40C	0.9700
C18—N5	1.556 (5)	C40—H40D	0.9700
C18—H18A	0.9700	C41—C42	1.471 (10)
C18—H18B	0.9700	C41—H41A	0.9700
C19—C20	1.560 (7)	C41—H41B	0.9700
C19—C20'	1.587 (10)	C42—H42A	0.9600
C19—H19A	0.9700	C42—H42B	0.9600
C19—H19B	0.9700	C42—H42C	0.9600
C19—H19C	0.9700	C20'—C21'	1.525 (10)
C19—H19D	0.9700	C20'—H20C	0.9700
C20—C21	1.486 (8)	C20'—H20D	0.9700
C20—H20A	0.9700	C21'—H21D	0.9600
C20—H20B	0.9700	C21'—H21E	0.9600
C21—H21A	0.9600	C21'—H21F	0.9600
C21—H21B	0.9600	C41'—C42'	1.499 (10)
C21—H21C	0.9600	C41'—H41C	0.9700
C22—C23	1.373 (4)	C41'—H41D	0.9700
C22—C27	1.377 (5)	C42'—H42D	0.9600
C22—N6	1.432 (4)	C42'—H42E	0.9600
C23—C24	1.387 (5)	C42'—H42F	0.9600
C23—H23	0.9300	N1—N2	1.384 (4)
C24—C25	1.372 (5)	N2—N3	1.298 (4)
C24—H24	0.9300	N6—N7	1.379 (3)
C25—C26	1.361 (5)	N7—N8	1.292 (4)
			
C6—C1—C2	119.8 (4)	N8—C29—C30	129.9 (3)
C6—C1—N1	121.5 (3)	O3—C30—C29	129.1 (4)
C2—C1—N1	118.8 (3)	O3—C30—N10	120.7 (4)
C3—C2—C1	119.6 (4)	C29—C30—N10	110.2 (3)
C3—C2—H2	120.2	N9—C31—O4	120.8 (3)
C1—C2—H2	120.2	N9—C31—N10	127.0 (3)
C4—C3—C2	121.2 (4)	O4—C31—N10	112.2 (3)
C4—C3—H3	119.4	C37—C32—C33	120.5 (3)
C2—C3—H3	119.4	C37—C32—O4	117.0 (3)
C3—C4—C5	119.4 (4)	C33—C32—O4	122.0 (3)
C3—C4—H4	120.3	C34—C33—C32	119.3 (3)
C5—C4—H4	120.3	C34—C33—H33	120.3
C4—C5—C6	119.9 (4)	C32—C33—H33	120.3
C4—C5—H5	120.1	C33—C34—C35	121.0 (3)
C6—C5—H5	120.1	C33—C34—H34	119.5
C1—C6—C5	120.1 (3)	C35—C34—H34	119.5
C1—C6—H6	119.9	O6—C35—C36	124.8 (3)
C5—C6—H6	119.9	O6—C35—C34	116.4 (3)
C8—C7—N4	127.8 (3)	C36—C35—C34	118.8 (3)
C8—C7—N1	105.1 (3)	C35—C36—C37	120.5 (3)
N4—C7—N1	127.0 (3)	C35—C36—H36	119.7
C7—C8—N3	110.2 (3)	C37—C36—H36	119.7
C7—C8—C9	120.2 (3)	C32—C37—C36	119.8 (3)
N3—C8—C9	129.5 (3)	C32—C37—H37	120.1
O1—C9—N5	120.9 (4)	C36—C37—H37	120.1
O1—C9—C8	128.0 (4)	O6—C38—H38A	109.5
N5—C9—C8	111.0 (3)	O6—C38—H38B	109.5
N4—C10—O2	121.5 (3)	H38A—C38—H38B	109.5
N4—C10—N5	127.2 (3)	O6—C38—H38C	109.5
O2—C10—N5	111.3 (3)	H38A—C38—H38C	109.5
C12—C11—C16	121.4 (3)	H38B—C38—H38C	109.5
C12—C11—O2	116.8 (3)	C40—C39—N10	109.1 (4)
C16—C11—O2	121.2 (3)	C40—C39—H39A	109.9
C11—C12—C13	120.2 (3)	N10—C39—H39A	109.9
C11—C12—H12	119.9	C40—C39—H39B	109.9
C13—C12—H12	119.9	N10—C39—H39B	109.9
C12—C13—C14	119.3 (3)	H39A—C39—H39B	108.3
C12—C13—H13	120.3	C39—C40—C41'	97.0 (6)
C14—C13—H13	120.3	C39—C40—C41	129.1 (7)
O5—C14—C15	115.7 (3)	C41'—C40—C41	33.0 (6)
O5—C14—C13	124.6 (3)	C39—C40—H40A	105.0
C15—C14—C13	119.7 (3)	C41'—C40—H40A	112.3
C14—C15—C16	121.0 (3)	C41—C40—H40A	105.0
C14—C15—H15	119.5	C39—C40—H40B	105.0
C16—C15—H15	119.5	C41'—C40—H40B	128.6
C11—C16—C15	118.3 (3)	C41—C40—H40B	105.0
C11—C16—H16	120.9	H40A—C40—H40B	105.9
C15—C16—H16	120.9	C39—C40—H40C	111.7
O5—C17—H17A	109.5	C41'—C40—H40C	112.5
O5—C17—H17B	109.5	C41—C40—H40C	101.3
H17A—C17—H17B	109.5	H40A—C40—H40C	7.0
O5—C17—H17C	109.5	H40B—C40—H40C	101.4
H17A—C17—H17C	109.5	C39—C40—H40D	112.6
H17B—C17—H17C	109.5	C41'—C40—H40D	112.7
C19—C18—N5	109.9 (4)	C41—C40—H40D	89.7
C19—C18—H18A	109.7	H40A—C40—H40D	115.4
N5—C18—H18A	109.7	H40B—C40—H40D	16.0
C19—C18—H18B	109.7	H40C—C40—H40D	109.8
N5—C18—H18B	109.7	C42—C41—C40	107.2 (9)
H18A—C18—H18B	108.2	C42—C41—H41A	110.3
C18—C19—C20	114.6 (6)	C40—C41—H41A	110.3
C18—C19—C20'	89.4 (7)	C42—C41—H41B	110.3
C20—C19—C20'	28.8 (6)	C40—C41—H41B	110.3
C18—C19—H19A	108.6	H41A—C41—H41B	108.5
C20—C19—H19A	108.6	C21'—C20'—C19	105.0 (9)
C20'—C19—H19A	106.6	C21'—C20'—H20C	110.7
C18—C19—H19B	108.6	C19—C20'—H20C	110.7
C20—C19—H19B	108.6	C21'—C20'—H20D	110.7
C20'—C19—H19B	133.2	C19—C20'—H20D	110.7
H19A—C19—H19B	107.6	H20C—C20'—H20D	108.8
C18—C19—H19C	113.7	C20'—C21'—H21D	109.5
C20—C19—H19C	111.4	C20'—C21'—H21E	109.5
C20'—C19—H19C	113.5	H21D—C21'—H21E	109.5
H19A—C19—H19C	9.2	C20'—C21'—H21F	109.5
H19B—C19—H19C	98.5	H21D—C21'—H21F	109.5
C18—C19—H19D	113.7	H21E—C21'—H21F	109.5
C20—C19—H19D	90.2	C42'—C41'—C40	105.5 (8)
C20'—C19—H19D	114.1	C42'—C41'—H41C	110.6
H19A—C19—H19D	120.1	C40—C41'—H41C	110.6
H19B—C19—H19D	19.2	C42'—C41'—H41D	110.6
H19C—C19—H19D	111.0	C40—C41'—H41D	110.6
C21—C20—C19	107.0 (6)	H41C—C41'—H41D	108.8
C21—C20—H20A	110.3	C41'—C42'—H42D	109.5
C19—C20—H20A	110.3	C41'—C42'—H42E	109.5
C21—C20—H20B	110.3	H42D—C42'—H42E	109.5
C19—C20—H20B	110.3	C41'—C42'—H42F	109.5
H20A—C20—H20B	108.6	H42D—C42'—H42F	109.5
C23—C22—C27	119.8 (3)	H42E—C42'—H42F	109.5
C23—C22—N6	120.9 (3)	C7—N1—N2	108.1 (3)
C27—C22—N6	119.3 (3)	C7—N1—C1	132.5 (3)
C22—C23—C24	119.0 (3)	N2—N1—C1	119.4 (3)
C22—C23—H23	120.5	N3—N2—N1	108.7 (3)
C24—C23—H23	120.5	N2—N3—C8	107.8 (3)
C25—C24—C23	121.0 (3)	C10—N4—C7	111.3 (3)
C25—C24—H24	119.5	C10—N5—C9	122.4 (3)
C23—C24—H24	119.5	C10—N5—C18	120.5 (3)
C26—C25—C24	118.9 (3)	C9—N5—C18	116.7 (3)
C26—C25—H25	120.5	C28—N6—N7	108.7 (2)
C24—C25—H25	120.5	C28—N6—C22	131.7 (2)
C25—C26—C27	121.2 (4)	N7—N6—C22	119.5 (3)
C25—C26—H26	119.4	N8—N7—N6	108.7 (3)
C27—C26—H26	119.4	N7—N8—C29	108.0 (2)
C26—C27—C22	120.1 (3)	C31—N9—C28	111.8 (2)
C26—C27—H27	119.9	C31—N10—C30	122.8 (3)
C22—C27—H27	119.9	C31—N10—C39	120.7 (3)
N6—C28—N9	127.6 (2)	C30—N10—C39	116.3 (3)
N6—C28—C29	105.3 (3)	C10—O2—C11	121.3 (2)
N9—C28—C29	127.0 (3)	C31—O4—C32	120.4 (2)
C28—C29—N8	109.2 (3)	C14—O5—C17	118.2 (3)
C28—C29—C30	121.0 (3)	C35—O6—C38	117.6 (3)
			
C6—C1—C2—C3	−0.7 (5)	N4—C7—N1—N2	179.2 (3)
N1—C1—C2—C3	178.0 (3)	C8—C7—N1—C1	−178.7 (3)
C1—C2—C3—C4	1.1 (6)	N4—C7—N1—C1	0.4 (5)
C2—C3—C4—C5	−1.2 (6)	C6—C1—N1—C7	−13.3 (5)
C3—C4—C5—C6	0.8 (6)	C2—C1—N1—C7	168.0 (3)
C2—C1—C6—C5	0.3 (5)	C6—C1—N1—N2	168.0 (3)
N1—C1—C6—C5	−178.3 (3)	C2—C1—N1—N2	−10.6 (4)
C4—C5—C6—C1	−0.4 (5)	C7—N1—N2—N3	0.2 (3)
N4—C7—C8—N3	−179.4 (3)	C1—N1—N2—N3	179.1 (3)
N1—C7—C8—N3	−0.3 (3)	N1—N2—N3—C8	−0.4 (4)
N4—C7—C8—C9	−0.8 (5)	C7—C8—N3—N2	0.4 (4)
N1—C7—C8—C9	178.3 (3)	C9—C8—N3—N2	−178.0 (3)
C7—C8—C9—O1	−178.8 (3)	O2—C10—N4—C7	178.2 (3)
N3—C8—C9—O1	−0.5 (6)	N5—C10—N4—C7	−0.2 (4)
C7—C8—C9—N5	0.2 (4)	C8—C7—N4—C10	0.8 (4)
N3—C8—C9—N5	178.5 (3)	N1—C7—N4—C10	−178.1 (3)
C16—C11—C12—C13	1.6 (5)	N4—C10—N5—C9	−0.4 (5)
O2—C11—C12—C13	−170.2 (3)	O2—C10—N5—C9	−178.9 (3)
C11—C12—C13—C14	−0.2 (4)	N4—C10—N5—C18	172.7 (3)
C12—C13—C14—O5	179.2 (3)	O2—C10—N5—C18	−5.9 (5)
C12—C13—C14—C15	−1.7 (5)	O1—C9—N5—C10	179.4 (3)
O5—C14—C15—C16	−178.5 (3)	C8—C9—N5—C10	0.3 (5)
C13—C14—C15—C16	2.3 (5)	O1—C9—N5—C18	6.1 (5)
C12—C11—C16—C15	−1.0 (5)	C8—C9—N5—C18	−173.0 (3)
O2—C11—C16—C15	170.4 (3)	C19—C18—N5—C10	96.9 (4)
C14—C15—C16—C11	−1.0 (5)	C19—C18—N5—C9	−89.7 (4)
N5—C18—C19—C20	−175.0 (4)	N9—C28—N6—N7	−178.9 (2)
N5—C18—C19—C20'	170.7 (10)	C29—C28—N6—N7	−0.1 (3)
C18—C19—C20—C21	−72.3 (10)	N9—C28—N6—C22	−0.8 (4)
C20'—C19—C20—C21	−41 (2)	C29—C28—N6—C22	178.0 (2)
C27—C22—C23—C24	0.9 (4)	C23—C22—N6—C28	10.2 (4)
N6—C22—C23—C24	−179.4 (3)	C27—C22—N6—C28	−170.0 (3)
C22—C23—C24—C25	−0.4 (5)	C23—C22—N6—N7	−171.8 (2)
C23—C24—C25—C26	−0.3 (5)	C27—C22—N6—N7	7.9 (4)
C24—C25—C26—C27	0.5 (6)	C28—N6—N7—N8	0.1 (3)
C25—C26—C27—C22	−0.1 (6)	C22—N6—N7—N8	−178.3 (2)
C23—C22—C27—C26	−0.7 (5)	N6—N7—N8—C29	0.0 (3)
N6—C22—C27—C26	179.6 (3)	C28—C29—N8—N7	−0.1 (3)
N6—C28—C29—N8	0.1 (3)	C30—C29—N8—N7	−179.2 (3)
N9—C28—C29—N8	178.9 (2)	O4—C31—N9—C28	−177.3 (3)
N6—C28—C29—C30	179.3 (3)	N10—C31—N9—C28	1.1 (4)
N9—C28—C29—C30	−1.9 (4)	N6—C28—N9—C31	−179.1 (3)
C28—C29—C30—O3	177.9 (3)	C29—C28—N9—C31	2.4 (4)
N8—C29—C30—O3	−3.1 (6)	N9—C31—N10—C30	−5.1 (5)
C28—C29—C30—N10	−1.8 (4)	O4—C31—N10—C30	173.4 (3)
N8—C29—C30—N10	177.2 (3)	N9—C31—N10—C39	169.6 (4)
C37—C32—C33—C34	1.7 (5)	O4—C31—N10—C39	−11.9 (5)
O4—C32—C33—C34	−170.3 (3)	O3—C30—N10—C31	−174.8 (3)
C32—C33—C34—C35	0.9 (5)	C29—C30—N10—C31	5.0 (4)
C33—C34—C35—O6	176.0 (3)	O3—C30—N10—C39	10.3 (5)
C33—C34—C35—C36	−2.8 (5)	C29—C30—N10—C39	−169.9 (4)
O6—C35—C36—C37	−176.5 (3)	C40—C39—N10—C31	91.3 (5)
C34—C35—C36—C37	2.3 (5)	C40—C39—N10—C30	−93.7 (5)
C33—C32—C37—C36	−2.2 (5)	N4—C10—O2—C11	8.4 (5)
O4—C32—C37—C36	170.1 (3)	N5—C10—O2—C11	−173.0 (3)
C35—C36—C37—C32	0.2 (5)	C12—C11—O2—C10	−132.0 (3)
N10—C39—C40—C41'	170.7 (7)	C16—C11—O2—C10	56.2 (4)
N10—C39—C40—C41	179.6 (11)	N9—C31—O4—C32	−7.8 (5)
C39—C40—C41—C42	−25 (2)	N10—C31—O4—C32	173.6 (3)
C41'—C40—C41—C42	−9.0 (16)	C37—C32—O4—C31	128.2 (3)
C18—C19—C20'—C21'	174.3 (17)	C33—C32—O4—C31	−59.6 (4)
C20—C19—C20'—C21'	22.1 (13)	C15—C14—O5—C17	166.1 (3)
C39—C40—C41'—C42'	−177.3 (11)	C13—C14—O5—C17	−14.8 (5)
C41—C40—C41'—C42'	15.4 (17)	C36—C35—O6—C38	11.9 (5)
C8—C7—N1—N2	0.1 (3)	C34—C35—O6—C38	−166.9 (4)
